# Plasticity engineering of plant monoterpene synthases and application for microbial production of monoterpenoids

**DOI:** 10.1186/s13068-021-01998-8

**Published:** 2021-06-30

**Authors:** Dengwei Lei, Zetian Qiu, Jianjun Qiao, Guang-Rong Zhao

**Affiliations:** 1 Frontier Science Center for Synthetic Biology and Key Laboratory of Systems Bioengineering (Ministry of Education), School of Chemical Engineering and Technology, Tianjin University, Yaguan Road 135, Jinnan District, Tianjin, 300350 China; 2 SynBio Research Platform, Collaborative Innovation Centre of Chemical Science and Engineering (Tianjin), Tianjin University, Yaguan Road 135, Jinnan District, Tianjin, 300350 China

**Keywords:** Monoterpene synthase, Functional plasticity, Synthetic biology, Enzyme engineering, Substrate selectivity, Product specificity, Monoterpenoid production

## Abstract

Plant monoterpenoids with structural diversities have extensive applications in food, cosmetics, pharmaceuticals, and biofuels. Due to the strong dependence on the geographical locations and seasonal annual growth of plants, agricultural production for monoterpenoids is less effective. Chemical synthesis is also uneconomic because of its high cost and pollution. Recently, emerging synthetic biology enables engineered microbes to possess great potential for the production of plant monoterpenoids. Both acyclic and cyclic monoterpenoids have been synthesized from fermentative sugars through heterologously reconstructing monoterpenoid biosynthetic pathways in microbes. Acting as catalytic templates, plant monoterpene synthases (MTPSs) take elaborate control of the monoterpenoids production. Most plant MTPSs have broad substrate or product properties, and show functional plasticity. Thus, the substrate selectivity, product outcomes, or enzymatic activities can be achieved by the active site mutations and domain swapping of plant MTPSs. This makes plasticity engineering a promising way to engineer MTPSs for efficient production of natural and non-natural monoterpenoids in microbial cell factories. Here, this review summarizes the key advances in plasticity engineering of plant MTPSs, including the fundamental aspects of functional plasticity, the utilization of natural and non-natural substrates, and the outcomes from product isomers to complexity-divergent monoterpenoids. Furthermore, the applications of plasticity engineering for improving monoterpenoids production in microbes are addressed.

**Supplementary Information:**

The online version contains supplementary material available at 10.1186/s13068-021-01998-8.

## Background

Monoterpenoids are members of the terpenoids superfamily with more than 11,000 monoterpenes and their derivatives identified [1]. Monoterpenoids have been extensively applied in food, cosmetics, agricultural, medicinal, and energy industries (Additional file 1: Table S1). Myrcene, linalool, geraniol, nerol, limonene, β-phellandrene, α-terpineol, and borneol are widely used as flavors or fragrances in foods, perfumes, and household products [2–6]. Geraniol and limonene have been developed as insecticides for agricultural protection [7]. Limonene has also been used for the treatment of cholecystitis and angiocholitis in clinics [8]. Paeoniflorin, a glycosylated derivative of limonene, possesses analgesic, sedative, anticonvulsant, anti-inflammatory, and neuroprotection properties, and has potential application in treating ischemic strokes [9]. Borneol is used as an analgesic drug for treating burns, wounds, cuts, and injuries, and as an indispensable ingredient of traditional Chinese medicines for cardiovascular diseases, including stroke, angina pectoris, and coronary heart disease [10]. Moreover, monoterpenoids can be exploited as biofuels. β-Phellandrene and sabinene can be gasoline alternatives for their high energy contents, low hygroscopicity and relatively low volatility [11, 12]. Likewise, pinene dimers, camphene dimers, and limonene dimers show high density and heating value comparable to the petroleum-based jet fuel JP-10 and can therefore be used as advanced biofuels [13]. Furthermore, linalool and 1,8-cineole can be used to produce aircraft fuels RJ-4 and AMJ-700, respectively [14, 15].

**Fig. 1:**
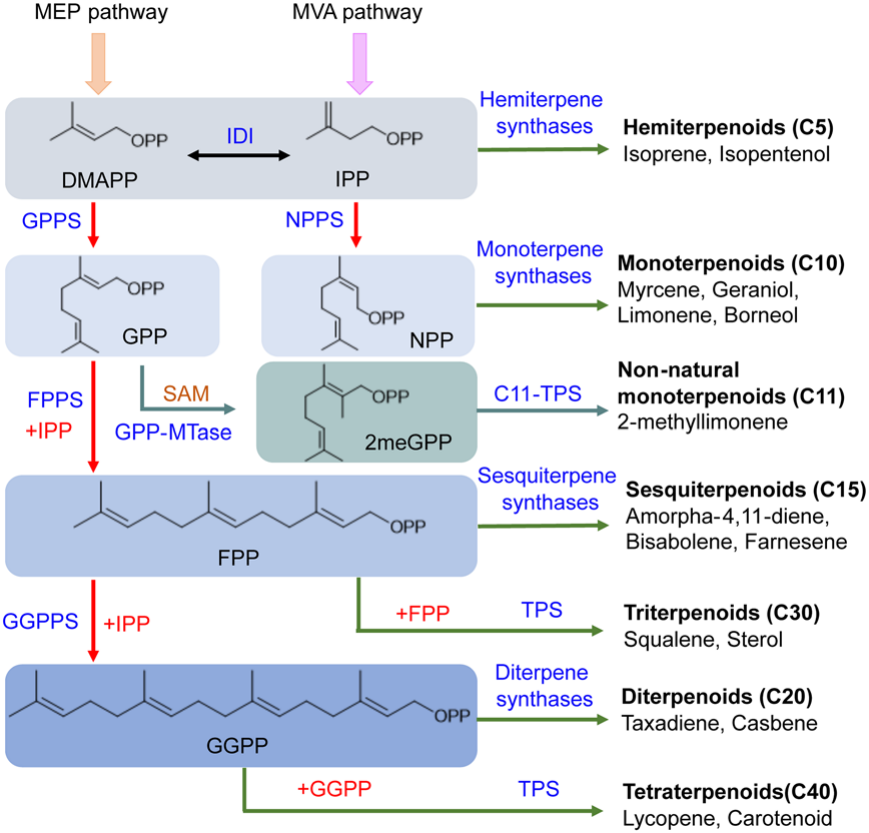
The biosynthetic pathway of terpenoids

All terpenoids (including monoterpenoids) utilize two C5 units isopentenyl diphosphate (IPP) and dimethylallyl diphosphate (DMAPP) as building blocks (Fig. 1), which can be provided by either the methylerythritol-4-phosphate (MEP) pathway or mevalonate (MVA) pathway [16]. IPP and DMAPP can be condensed by various prenyltransferases to generate an array of prenyl diphosphate precursors of different chain lengths, including geranyl diphosphate (GPP, trans-isomer; C10), neryl diphosphate (NPP, cis-isomer; C10), farnesyl diphosphate (FPP; C15), and geranylgeranyl diphosphate (GGPP; C20). These prenyl precursors are then harnessed by a series of terpene synthases (TPSs) to produce hemiterpenoids, monoterpenoids, sesquiterpenoids, diterpenoids, triterpenoids, or tetraterpenoids (Fig. 1). Especially, non-natural C11-terpenoids can be synthesized from the C11-substrate 2-methyl-GPP (2meGPP), which is derived from GPP by GPP methyltransferase (GPP-MTase) [17, 18].

Agricultural production can be an approach to supply monoterpenoids, but it is less effective on an industrial scale due to the strong dependence on the geographical locations and seasonal annual growth of plants [19]. Similarly, the chemical synthesis of monoterpenoids is a high-cost and less environmentally friendly option on account of the complex reactions, high energy consumption, and serious pollutions [6, 20]. Microbial production of monoterpenoids, such as using *Escherichia coli* and yeast as cell factories, has great potential for meeting the huge market demands [16, 21]. To date, 24 g/L isoprene [22], 40 g/L amorphadiene [23], and 130 g/L farnesene [24] have been reported in engineered microbes. However, the microbial production of monoterpenoids falls far behind hemiterpenoids and sesquiterpenoids. Except for the gram-scale titers of geraniol (5.52 g/L) [25], linalool (5.60 g/L) [26], limonene (3.63 g/L) [27], and sabinene (2.65 g/L) [28] being achieved, other monoterpenoids are in the milligram-scale production [6, 29, 30].

In the biosynthesis of monoterpenoids, monoterpene synthases (MTPSs) act as the key machine, determining the reaction rate and ultimate product profiles to a large extent [29]. A majority of plant MTPSs exhibit functional plasticity with changes of substrate or product properties [31, 32]. Plasticity engineering of plant MTPSs evolves the substrate selectivity, product specificity, and catalytic activity by small modifications to improve monoterpenoids production in microbial cell factories. Herein, we review the plasticity engineering of plant MTPSs for the efficient production of monoterpenoids in microbes. We firstly highlight the tightly linked reaction sequences of various monoterpenoids and the typical structural features of plant MTPSs to elucidate the chemical and structural fundamentals of functional plasticity. Meanwhile, we provide a brief overview of the function-sensitive plasticity regions of plant MTPSs. Further, we pay special attention to the plasticity engineering of plant MTPSs for altering substrate utilization and product specificity, respectively. We finally portray the applications of plasticity engineering of plant MTPSs for the microbial synthesis of monoterpenoids.

## Fundamentals of the functional plasticity of plant MTPSs

### Tightly associated reaction cascades for products formation

**Fig. 2:**
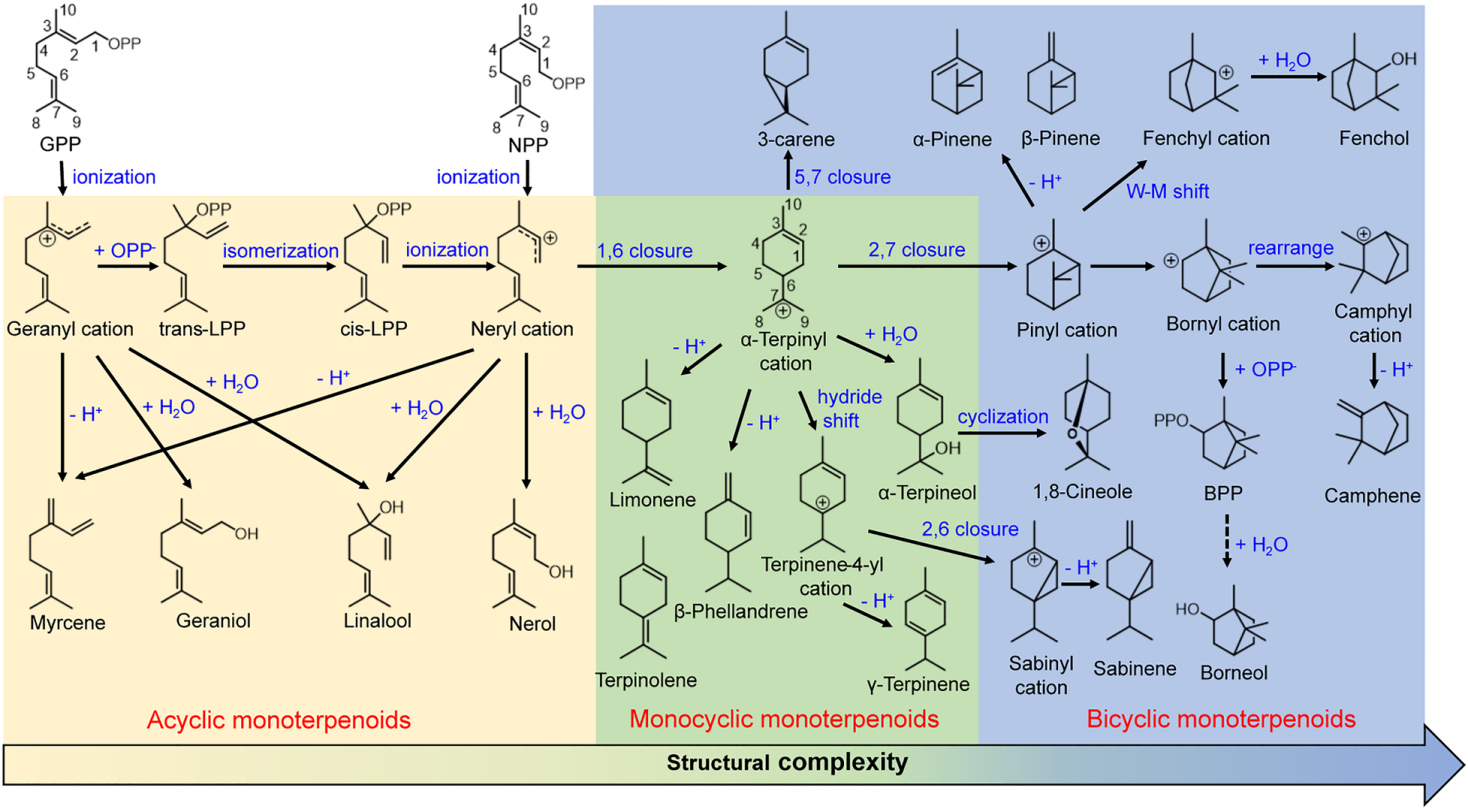
The monoterpenoid reaction cascades catalyzed by MTPSs

Plant MTPSs undergo various but closely linked reaction cascades to output the end products, as shown in Fig. 2. Generally, GPP serves as the precursor of monoterpenoids. The diphosphate group of GPP coordinates with the conserved motifs of MTPS and three metal ions (generally Mn^2+^ or Mg^2+^) to form a trinary complex of enzyme–substrate–cofactors, triggering the ionization of GPP [33]. GPP thus removes the diphosphate and generates the first carbocation (geranyl cation), which can form acyclic myrcene via deprotonation by myrcene synthase (MyrS) [34], or acyclic monoterpene alcohols such as geraniol by geraniol synthase (GerS) and linalool by linalool synthase (LinS) via the addition of one water molecule [35], whereas in the reaction cascades of cyclic MTPSs, the carbon bond between C2 and C3 is required to rotate to cisoid conformation before the cyclization process [36]. After the first ionization, the geranyl cation rebinds a diphosphate group to C3, synthesizing trans-linalyl diphosphate (trans-LPP), and further isomerizes to the cis-LPP. The cis-LPP undergoes the second ionization, generating the neryl cation, which can lead to acyclic monoterpenoids by acyclic MTPSs including nerol synthase (NerS) [37]. Notably, the cisoid substrate NPP can be ionized directly to form the neryl cation without the isomerization process [38]. The neryl cation further undergoes 1,6-ring closure to the pivotal carbocation intermediate (α-terpinyl cation), which then gives rise to monocyclic or bicyclic monoterpenoids [36] (Fig. 2). The terpinyl cation can be quenched through deprotonation, resulting in a plethora of monocyclic monoterpenes, e.g., limonene, terpinolene, and β-phellandrene. Also, the terpinyl cation can be transformed into terpinene-4-yl cation by γ-terpinene synthase (TerS) through the 6,7-hydride shift, and finally yields terpinene after proton elimination [39]. When the α-terpinyl cation is captured by one water molecular by terpineol synthase (TepS), it eventually generates terpineol [40].

Bicyclic MTPSs take the α-terpinyl cation as a hinge of cyclization cascades as well (Fig. 2). The terpinyl cation undergoes a second cyclization between diverse carbon atoms to form various bicyclic carbocations. The 5,7-ring closure by 3-carene synthase (CarS) gives rise to 3-carene [41]. Whereas the pinyl cation is generated from the 2,7-ring closure of the terpinyl cation, producing α-/β-pinene via deprotonation by pinene synthase (PinS) [42]. Furthermore, the pinyl cation can proceed to generate the fenchyl cation through Wagner–Meerwein rearrangement (W-M shift) by fenchol synthase (FenS), which is attacked by water molecules to generate fenchol [42]. Notably, the bornyl cation probably results from the pinyl cation and turns out to be another bifurcation point of the monoterpenoid catalytic cascades [43]. The bornyl cation can either be recaptured by diphosphate anion to produce bornyl diphosphate (BPP) by BPP synthase (BPPS) and then gives rise to borneol after pyrophosphate hydrolysis by hydrolases, or rearrange to the camphyl cation by camphene synthase (CamS) resulting in the synthesis of camphene [43]. In particular, the bicyclic sabinene is derived from the deprotonation of the sabinyl cation, which is the result of 2,6-ring closure of the terpinene-4-yl cation by sabinene synthase (SabS) [41]. As with 1,8-cineole synthase (CinS), bicyclic cineole distinctively comes from α-terpineol via protonation of the endocyclic double bond and cyclization [44].

### Typical structural features of plant MTPSs

**Fig. 3:**
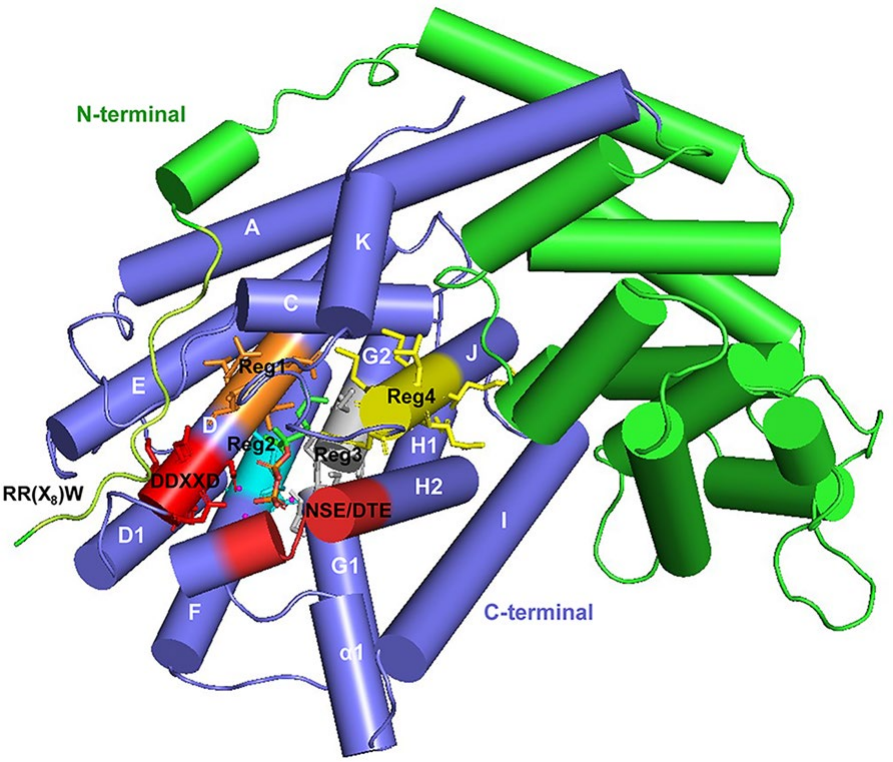
The structure and plasticity regions of plant MTPSs. The structure and plasticity regions of plant monoterpene synthases are based on the limonene synthases from *Mentha spicata* with substrate analog FGPP (PDB: 2ong) [45]. The N-terminal and C-terminal domains are shown in green and purple, respectively. The metal cofactors (Mn^2+^) are indicated in magenta, while the two metal-binding motifs (DDXXD and NSE/DTE motifs) are highlighted in red. The RR(X_8_)W motif on the N-terminal domain is distinguished in lemon. The four plasticity regions (Reg1–4 corresponding to regions 1–4) are highlighted in orange, cyan, gray and yellow, respectively.

The whole reaction sequence of each MTPS is under the precise control of the active sites, from the recognization of substrate to the formation of end products. Therefore, the active site contours of plant MTPSs are generally product-like, directing the generation of specific monoterpenoids [33]. Plant MTPSs regularly contain 600–650 amino acids, 50–70 amino acids longer than sesquiterpene synthases owing to the transit peptide sequence for plastidial (chloroplast) localization, and more than 200 amino acids shorter than diterpene synthases for fewer conserved motifs [30]. On the tertiary structure, plant MPTSs generally adopt a two-domain architecture, which is composed of N-terminal and C-terminal domains [33, 45] (Fig. 3). The C-terminal domain of MTPS presents as orthogonal bundles containing 12 helices and is responsible for the catalytic functions [46, 47]. However, the N-terminal domain of plant MTPSs presents as α/α barrel, and its function remains unclear. The active site cavity of plant MTPSs exploits six helices (C, D, F, G1-G2, H2-H1-α1, and J) of the C-terminal domain as walls (Fig. 3), which are composed of predominantly nonpolar (including hydrophobic and aromatic) amino acid residues, stabilizing carbocations through interactions with the carbon chain [46, 47]. Furthermore, the active site cavity is flanked by two metal cofactor binding motifs on helices D and H2 (Fig. 3). The first metal-binding motif, the aspartate-rich DDXXD motif on helix D, is highly conserved among class I terpenoid synthases (which generate initial carbocation intermediates by metal-dependent ionization), including monoterpene synthases, sesquiterpene synthases and part of diterpene synthases [33]. The other conserved motif coordinating with metal ions presents as (N/D)D(L/I/V)X(S/T)XXXE on helix H2, which is designated as NSE/DTE motif.

In the cyclization of monoterpenoids, two characteristic motifs of plant MTPSs are crucial. The first one is the RR(X_8_)W motif on the N-terminal strand (Fig. 3), which may be indirectly involved in the isomerization of GPP to cis-LPP [40]. For example, the limonene synthase (LimS) of *Mentha spicata* (MsLimS) possesses a flexible structure in the active sites to accommodate different prenyl diphosphates (e.g., GPP and cis-LPP) on the reaction cascades [45]. This is likely associated with the weak interactions between the tandem arginine residues and the C-terminal residues. The second motif lies 35 amino acids upstream of the DDXXD motif, namely the RXR motif, which is part of the active sites and facilitates the secondary ionization and cyclization in the generation of bicyclic monoterpenoids [40]. Moreover, MTPSs may adopt a productive conformation in the reaction process. The tandem arginine residues on the N-terminal strand of MsLimS lock the active site cavity to form a closed conformation after the isomerization of GPP to finally produce limonene (Fig. 3). Similarly, the *Citrus sinensis* LimS (CsLimS) and *Salvia officinalis* BPPS (SoBPPS) change to closed conformations from open conformations to exclude bulk solvent after the binding of substrate [46–48]. Also, it is vital for terpenoid cyclases to precisely control the trapped water molecule to output the desired products [33]. In the SoBPPS, one trapped water molecule in the active site cavity forms hydrogen bonds with the diphosphate group and residues Y426 and S451 [46]. The water molecule can serve as a diphosphate-assisted general base and facilitate the direct deprotonation of carbocation intermediates [46].

### Plasticity regions in the C-terminal domain

**Fig. 4:**
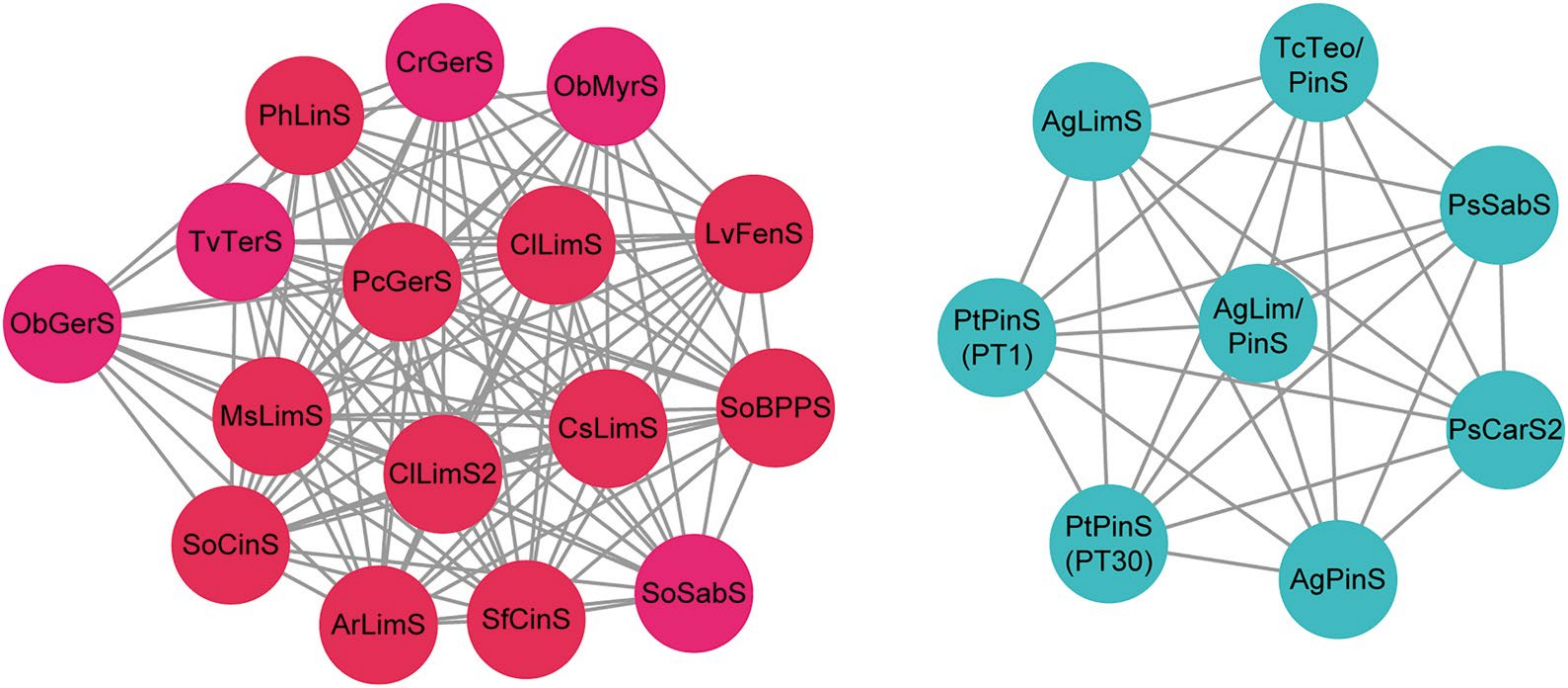
The sequence similarity network (SSN) of plant MTPSs discussed in this review. The SSN was generated using the EFI-EST web tool (https://efi.igb.illinois.edu/efi-est) with an alignment score threshold of 90.

The sequence similarity network (SSN) of plant MTPSs that have been engineered (Additional file 1: Table S2) presents two separate clusters (Fig. 4). Except for acyclic MTPSs being deposited in the one cluster, the monocyclic and bicyclic MTPSs are not grouped individually. The mismatch between the sequence similarity and the functions of plant MTPSs in the SSN indicates that different plant MTPSs may share a high protein identity and that their catalytic functions can be modified through plasticity engineering.

**Fig. 5:**
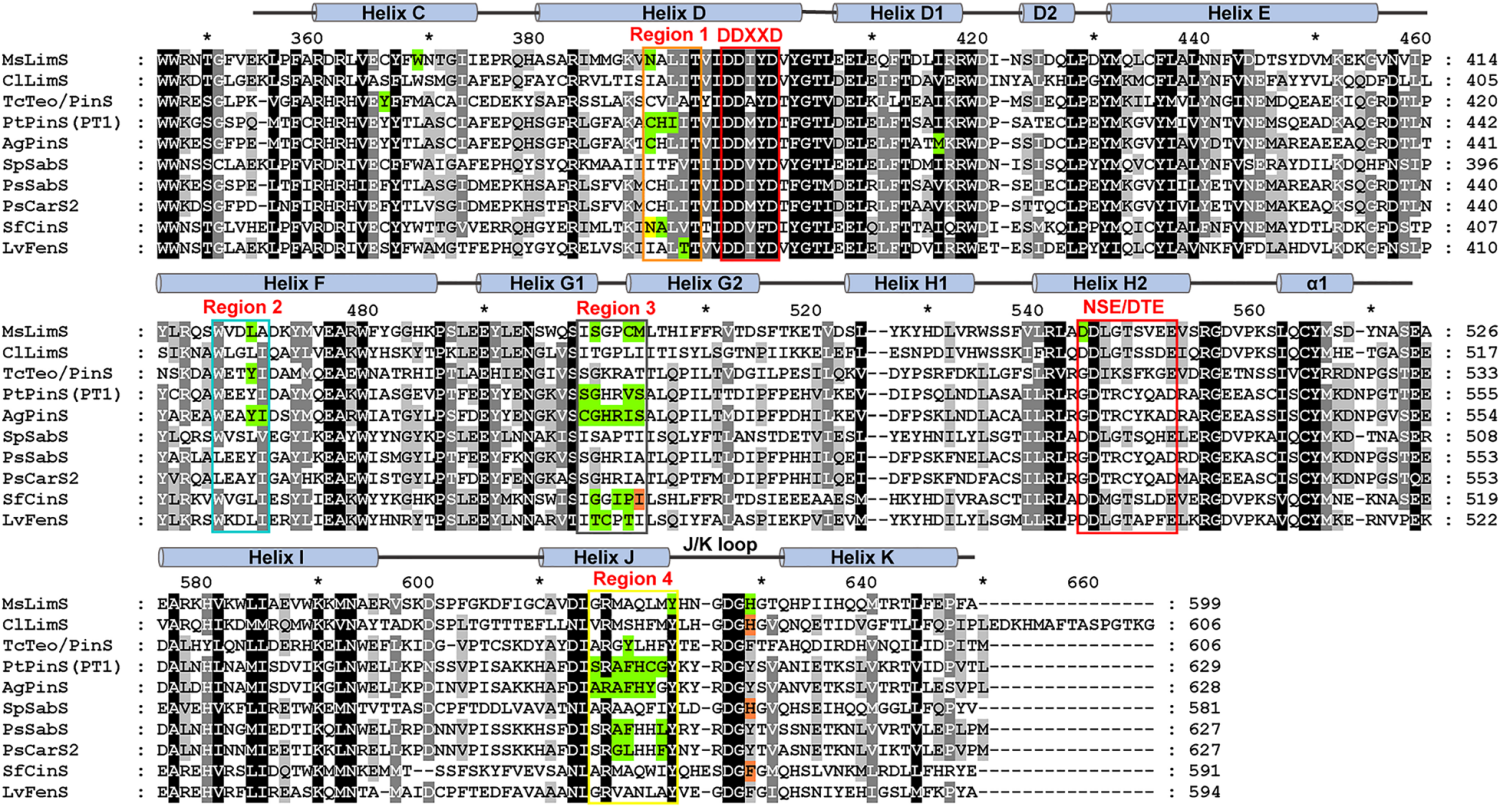
Functional plasticity residues and regions in the C-terminal domain of plant MTPSs. The two metal-binding motifs (DDXXD and NSE/DTE motifs) and the four plasticity regions (Regions 1–4) are framed in the same colors as Fig. 3. Residues affecting substrate selectivity and product specificity are, respectively, highlighted in orange and green background, while residues affecting both substrate and product properties are highlighted in yellow background.

Enzyme engineering shows that plant MTPSs embrace the majority of function-sensitive plasticity residues in the C-terminal domain, which have been clustered into four plasticity regions by Leferink et al. [32], along with a few plasticity residues scattering out of these regions (Figs. 3 and  5). The first plasticity region (Region 1) consists of five residues, which locates two amino acids upstream of the conserved DDXXD motif on helix D (Fig. 5). Residues in Region 1 can contribute to enlarging the binding pocket, stabilizing the carbocation intermediates, or facilitating the deprotonation of the α-terpinyl cation [32, 44]. The second plasticity region (Region 2) on helix F involves five residues, which may affect the conformation of the active site cavity or affect the deprotonation of the terpinyl cation through steric constrains [49, 50]. The third plasticity region (Region 3) includes six residues spanning helices G1 and G2 (Fig. 5). Residues in Region 3 are thought to play important roles in the generation and stabilization of cation intermediates in the early stages of reaction cascades [32, 44]. The last plasticity region (Region 4) contains eight residues of helix J at the end of the C-terminal domain (Fig. 5). These residues may be involved in capping the active site cavity to exclude bulk solvent (Fig. 3) and stabilizing more complex carbocations [32, 46–48]. Consequently, residues in these plasticity regions have shown complex effects on substrate selectivity, product profiles, and catalytic activity of plant MTPSs (Fig. 5).

## Plasticity engineering of plant MTPSs for substrate utilization

GPP is the canonical substrate for plant MTPSs to synthesize monoterpenoids in planta. More and more evidence shows that NPP can be used as an alternative substrate by plant MPTSs [37, 38, 51]. Furthermore, some plant MPTSs can utilize FPP and GGPP as substrates [52–55]. Certain residues in the active site pocket may act as determinants of the substrate selectivity of MTPSs (Fig. 5). Hence, the substrate utilization of plant MTPSs toward isoprenoid diphosphates can be altered by modifying these functional plasticity residues and are summarized in Table 1.

**Table 1:** Representative examples of plasticity engineering of plant MTPSs for altering substrate utilization

Plasticity about	Plant MTPSs	Wild type/variants	Positions of plasticity residues	Substrate utilization
C10-isomers GPP/NPP [31]	SfCinS	Wild type	–	71% lower *K*_M_ value and 55% higher efficiency for GPP than NPP
		F571H	J/K loop	92% lower *K*_M_ value and 1500% higher efficiency for GPP than NPP
		F571V	J/K loop	75% lower *K*_M_ value and 600% higher efficiency for NPP than GPP
	ClLimS	Wild type	–	69% lower *K*_M_ value and 61% higher efficiency for GPP than NPP
		H570V	J/K loop	43% lower *K*_M_ value and 81% higher efficiency for NPP than GPP
		H570I	J/K loop	9% lower *K*_M_ value and 678% higher efficiency for NPP than GPP
	SeCamS	Wild type	–	83% lower *K*_M_ value and 377% higher efficiency for GPP than NPP
		H583F/ H583V	J/K loop	~ 200% higher selectivity for NPP than the wild type
GPP/FPP [44]	SfCinS	Wild type	–	GPP-specific without activity to FPP
		N338A	Region 1	84% lower *K*_M_ value and 18,216% higher efficiency for GPP than FPP
		N338S	Region 1	54% lower *K*_M_ value and 11,250% higher efficiency for GPP than FPP
		N338C	Region 1	50% lower *K*_M_ value for FPP than GPP
Natural GPP/non-natural 2meGPP [61]	SfCinS	Wild type	–	36% lower *K*_M_ value and 530% higher efficiency for GPP than 2meGPP
		N338S-I451A	Region 1 (N338), Region 3 (I451)	66% lower *K*_M_ value but 14% lower efficiency for 2meGPP than GPP
		F571Y	J/K loop	58% lower *K*_M_ value and 28% higher efficiency for 2meGPP than GPP
	ClLimS	Wild type	–	32% higher *K*_M_ value but 2500% higher efficiency for GPP than 2me GPP
		H570V	J/K loop	47% lower *K*_M_ value and 27% higher efficiency for 2meGPP than GPP
		H570L	J/K loop	16% lower *K*_M_ value and 58% higher efficiency for 2meGPP than GPP
		H570I	J/K loop	22% lower *K*_M_ value and 123% higher efficiency for 2meGPP than GPP
	SeCamS	Wild type	–	85% lower *K*_M_ value and 16,990% higher efficiency for GPP than 2meGPP
		H583L	J/K loop	41% lower *K*_M_ value and 8% higher efficiency for 2meGPP than GPP

Efficiency represents the *k*_cat_/*K*_M_ value. Regions 1–4 are corresponding to plasticity regions 1–4

### Altering substrate selectivity for GPP or NPP

Although monoterpene synthases can utilize the transoid GPP and the cisoid NPP (Fig. 1), generally most natural plant MTPSs prefer GPP to NPP as the substrate [56–58]. However, the substrate selectivity of the *Salvia fruticosa* CinS (SfCinS) was dramatically altered by modifying residue F571, a scattered plasticity residue on the J/K loop (Table 1). Variant SfCinS^F571H^ enhanced the selectivity for GPP by significantly reducing the affinity for NPP (with a 170% increase of *K*_M_ value), while variant SfCinS^F571V^ became NPP-specific as the *K*_M_ value and efficiency (*k*_cat_/*K*_M_) for NPP were 25% and 700% of those for GPP, respectively [31]. The geometry of the active site cavity may be changed by modifying residue 571, which is in proximity to the C1 atom of LPP, to favor the binding of either GPP or NPP [31]. Similarly, mutations of the corresponding plasticity residues in the *Citrus limon* LimS (ClLimS) and *Solanum elaeagnifolium* CamS (SeCamS) also improved the substrate selectivity for NPP [31]. This further highlights the plasticity of the site corresponding to residue 571 of SfCinS on selecting substrates in plant MTPSs.

### Accepting FPP as substrate

Plasticity engineering also allows plant MTPSs to accept length-diverse prenyl substrates via substitutions of plasticity residues (Table 1). Isoprene synthases have two conserved Phe residues (corresponding to residues F338 and F485 in the *Populus alba* isoprene synthase), both of which are considered to hinder C10 substrates from binding by reducing the size of the cavity [59]. Lacking the second Phe residue in the active site pocket, the *Humulus lupulus* MyrS (HlMyrS) showed to be a bifunctional enzyme and could accept both DMAPP and GPP as substrates [59, 60]. Therefore, plasticity engineering to modify the volume of the active site pocket is effective for changing the substrate tolerance of plant MTPSs. Substituting residue N338 in Region 1 of the SfCinS with less bulky amino acids to enlarge the active site cavity, enabled SfCinS mutants to accept longer FPP as substrate, albeit these mutants remained the activity to GPP [44]. In particular, variant SfCinS^N338C^ exhibited a higher affinity for FPP, with a half lower *K*_M_ value, compared to GPP [44]. Substitution of residue 338 results in the removal of the associated water molecule and meanwhile decreases the volume of the side chain, which provide a larger space of the active site cavity for the longer chain substrate FPP [44].

### Accommodating non-natural substrate

Plasticity engineering of plant MTPSs can shed light on the utilization of non-natural substrate to expand the kingdom of terpenoids (Table 1). Although the wild-type SfCinS showed stronger affinity and efficiency for GPP, it could also use non-natural C11 substrate 2meGPP in vitro and in vivo [61]. When introducing double mutation to enlarge the active site cavity, mutant SfCinS^N388S−I451A^ turned out to be 2meGPP-specific. The scattered plasticity residue F571 on the J/K loop of SfCinS possibly exerted an effect on the conversion of substrate conformation, and mutant SfCinS^F571Y^ increased the affinity for 2meGPP with a 58% lower *K*_M_ value than that for GPP [61]. Likewise, several dedicated 2meGPP-utilizing MTPSs have been developed through modifying this plasticity residue (homologous to F571 of SfCinS) in other plant MTPSs (Table 1), including SeCamS, ClLimS, *Ocimum basilicum* MyrS (ObMyrS), *Ocimum basilicum* GerS (ObGerS), and *Pinus taeda* PinS (PtPinS, PT30) [61].

## Plasticity engineering of plant MTPSs for product specificity

In nature, the products of plant MTPSs are structurally diverse and commonly exist in the differentiated cells or tissues of plants as a blend of monoterpenoids [62]. Even if some plant MTPSs share a high identity (more than 90%) of amino acid sequences, they can result in widely divergent product profiles [63–66]. Consistently, plasticity engineering of plant MTPSs is capable of changing products to a different degree, from product isomers to complexity-divergent monoterpenoids (Table 2).

**Table 2:** Representative examples of plasticity engineering of plant MTPSs for modifying product spectrum

Plasticity about	Plant MTPSs	Wild type/mutants	Positions of plasticity residues	Percentage of major products	Ref.
Product isomers	AgPinS	Wild type	-	29.5% α-pinene, 63.4% β-pinene	[49]
		C480S	Region 3	50.6% α-pinene, 41.5% β-pinene	
		S485C	Region 3	47.8% α-pinene, 43.3% β-pinene	
		F597W	Region 4	60.6% α-pinene, 29.4% β-pinene	
		C372S-C480S-S485C-F597W	Region 1 (C372), Region 3, and Region 4	79.5% α-pinene, 10.4% β-pinene	
Similar complexity	PhLinS	Wild type	–	100% linalool	[67]
		Exchanging domains IV-1 and IV-4 from PcGerS	covering Region 3 (domain IV-1) and Region 4 (domain IV-4)	100% geraniol	
	PcGerS	Wild type	–	100% geraniol	[67]
		Swapping domains III-b, III-d and IV-4 from PhLinS	covering Region 1 (domain III-b), Region 2 (domain III-d), and Region 4 (domain IV-4)	94% linalool	
	AgLimS	Wild type	–	71.9% limonene, 16.1% pinene, 11.3% phellandrene	[68]
		Introducing segment (position 375 to end) from AgLim/PinS	Covering regions 1–4	35.7% phellandrene, 28.5% limonene, 24.9% pinene	
		V384L	Region 1	64.9% limonene, 19.6% phellandrene, 15.5% pinene	
	SfCinS	Wild type	–	72.4% 1,8-cineole	[44]
		N338I	Region 1	48.3% sabinene, 37% limonene	
		N338V	Region 1	61.2% sabinene, 30.8% limonene	
		N338I-A339T- G447S-I449P- P450T	Region 1 (N338, A339) and Region 3 (G447, I449, P450)	86% sabinene	
	PsSabS	Wild type	–	44.7% sabinene, 35.9% terpinolene	[41]
		A595G-F596L- L599F	Region 4	42.3% 3-carene, 20% terpinolene	
	PsCarS2	Wild type	–	67.5% 3-carene, 15.4% terpinolene	[41]
		G595A-L596F- F599L	Region 4	47.4% sabinene, 35.2% terpinolene	
Divergent complexity	MsLimS	Wild type	–	96.6% limonene, 2.3% pinene,	[70]
		S454G	Region 3	52.13% limonene, 46.1% pinene	[71]
		N345A	Region 1	39.7% sabinene, 29.3% pinene, 23.3% limonene	[70]
		N345L	Region 1	51.1% pinene, 25.04% limonene, 21.83% phellandrene	[71]
		N345I	Region 1	68.87% phellandrene, 18.48% limonene, 11.79% pinene	[71]
		Y573F	Region 4	88.48% limonene, 4.91% pinene, 4.77% sabinene	[72]
		D496N	NSE/DTE motif	99.23% limonene	[72]
		W324Y/W324P	Helix C	~ 80% linalool	[70]
		H579A/H579D	J/K loop	~ 55% limonene, ~ 25% terpineol	[70]
		H579K/H579W	J/K loop	~ 40% limonene, ~ 25% linalool, 21%-26% terpineol	[70]
		M458A	Region 3	30.4% terpineol, 28.7% linalool, 11.8% limonene, 10.1% myrcene	[70]
	TcTeo/PinS	Wild type	–	Mainly α-pinene and terpinolene	[50]
		Y327F/ Y429F/ Y575F	Region 2 (Y429), Region 4 (Y575)	Terpinolene predominantly	
	PsSabS	Wild type	–	44.7% sabinene, 35.9% terpinolene	[41]
		F596E	Region 4	70.9% limonene, 10.2% sabinene	

The products of MTPSs in this table are derived from GPP. Regions 1–4 represent plasticity regions 1–4

### Refining product isomers

Plasticity engineering of plant MTPSs enables the refining of monoterpenoid isomers (Table 2). α-Pinene and β-pinene are two product isomers of PinS. The single point mutagenesis of C372S, C480S, S485C, or F597W in the *Abies grandis* PinS (AgPinS) resulted in the main product shift from β-pinene to α-pinene, while the four-point combination mutant of AgPinS further greatly increased the portion of α-pinene to 80% [49]. This may be linked with the influence of residues 480 and 485 in Region 3 as terminal proton acceptors and the effects of residue 372 in Region 1 and residue 597 in Region 4 on the orientation of pinyl cation intermediate [49].

### Switching monoterpenoids within similar complexity group

Monoterpenoids within a similar complexity group (Fig. 2) can be switched by plasticity engineering of plant MTPSs, and some excellent examples are listed in Table 2. Domain swapping between the *Perilla hirtella* LinS (PhLinS) and the *P. citriodora* GerS (PcGerS) led to acyclic products switch [67]. Exchanging domains IV-1 and IV-4 of PhLinS with those of PcGerS, the modified PhLinS became GerS and synthesized 100% geraniol. While swapping domains III-b, III-d, and IV-4 of PcGerS with those of PhLinS, the engineered PcGerS was switched to produce 94% linalool. Domains IV-1 (covering Region 3) and IV-4 (covering Region 4) are on the surface of the active site cavity in the modified PhLinS, whereas domains of III-b (covering Region 1), III-d (containing Region 2), and IV-4 (covering Region 4) surround the active site pocket in the engineered PcGerS. These crucial domains possibly influence the conformations of carbocation intermediates and their electron localization [67].

By domain swapping or point mutation of *A. grandis* LimS (AgLimS), the product was switched from limonene to β-phellandrene in the monocyclic monoterpenoid group [68]. By replacing the C-terminal segment of the AgLimS with that of *A. grandis* limonene/α-pinene synthase (AgLim/PinS), the modified AgLimS enhanced the ratio of β-phellandrene by 216%. Analogously, the single point mutation of V384L in Region 1 of AgLimS gave rise to almost double the amount of β-phellandrene with less limonene.

The product profiles of plant MTPSs can be transformed within the bicyclic monoterpenoid group. The product spectra of SoBPPS, SoCinS, and SoSabS were significantly altered by swapping segments of the C-terminal domain. The engineered SoSabS, which exchanged a segment (from position 304 to the C-terminal end) with the SoCinS, was switched to be a 1,8-cineole synthase [69]; whereas, swapping segments (residues 304 to 430 and residues 499 to the C-terminal end) from SoBPPS into SoSabS generated the modified SoSabS, which produced predominantly α-pinene without sabinene [69]. Most notably, the single substitution of N338I or N338V converted the SfCinS to be a sabinene synthase, and mutant SfCinS^N338I−A339T−G447S−I449P−P450T^ generated 86% sabinene without the formation of 1,8-cineole [44]. Residue N338 is probably critical for hydroxylating the terpinyl cation to form α-terpineol (Fig. 2), by the deprotonation effect of the hydrogen-bonding water molecule. Analogously, the product profiles of the *Picea sitchensis* SabS (PsSabS) and PsCarS2 can be switched by substitutions of residues 595, 596, and 599 in Region 4 [41] (Table 2). Furthermore, the AgPinS was changed to produce more than 50% bornyl cation-derived products (such as camphene) by combining multiple site mutations (C372S-Y450C-M398I-I451F) with the exchange of two segments (residues C480 to S485 in Region 3 and residues A594 to Y599 in Region 4) with the *A. grandis* CamS [49]. In addition to the effects of the active site configuration on the outcomes of products, some residues far away from the active center may play roles in monoterpenoids formation. Three residues (scattered plasticity residue M398 on helix D1 and residues Y450-I451 in Region 2) are not part of the active sites in AgPinS, but they show product plasticity as well [49]. Although the mechanisms are still unclear, it may be on account of the conformation changes of the active site pocket caused by substitutions of these residues [49].

### Changing products across divergent complexity groups

The product plasticity of plant MTPSs can be achieved across different complexity groups (Fig. 2), including plasticity between monocyclic and bicyclic products, and between cyclic and acyclic products by mutational genetic engineering (Table 2). The wild-type MsLimS produced about 97% limonene with very little pinene. Modifying residues N345, L423, S454, and Y573 (in Regions 1, 2, 3, and 4, respectively) of MsLimS resulted in a larger proportion of bicyclic products [70, 71]. The mutant MsLimS^S454G^ increased the ratio of pinene to 46% with a decrease of limonene, while the mutation of N345 in MsLimS led to increasing the biosynthesis of pinene as well as sabinene or phellandrene [70, 71]. Residues N345 and S454 may place steric constraints to hamper the 2,7-ring closure, therefore substituting them with less bulky residues can increase the production of bicyclic pinene [71].

Modifications of plasticity residues can contribute to a greater ratio of less complex products. Mutant MsLimS^D496N^ increased the ratio of limonene to 99% with 0.2% pinene [72]. Furthermore, the MsLimS was changed to generate more derailment products (such as acyclic myrcene, linalool, and monocyclic terpineol) by altering the scattered plasticity residues (W324 and H579) or residue M458 in Region 3 [70]. The mutation of W324 of MsLimS resulted in linalool as the major product, while the mutation of H579 of MsLimS increased the ratio of terpineol and linalool. Residues W324 and H579 of MsLimS probably stabilize the terpinyl cation and function as catalytic bases to assist the deprotonation process, thus showing strong functional plasticity [70].

The α-pinene/terpinolene synthase from *Taiwania cryptomerioides* (TcTeo/PinS) was shifted to produce terpinolene predominantly by the single site mutation of Y327F, Y429F, or Y575F (Table 2). Residues Y327 and Y429 in Region 2 of TcTeo/PinS may hamper the deprotonation of the terpinyl cation through steric hindrance, and thus their mutations do not favor the secondary cyclization process [50]. Moreover, the PsSabS was modified to produce 71% limonene by the single mutation of F596E, with the decrease of sabinene and terpinolene, which were the major products of the wild-type PsSabS (Table 2). Residue F596 in Region 4 of PsSabS is close to the terpinyl intermediate and favors the generation of sabinene for its stabilization effect on carbocation, which results from steric or van der Waals interactions [41].

## Microbial production of monoterpenoids

The substrate selectivity, product specificity, and catalytic activity of plant MTPSs can be tamed by modifying the regional or scattered functional plasticity residues. Therefore, plasticity engineering of plant MTPSs can be applied for microbial production of monoterpenoids, including exploiting the orthogonal monoterpenoid biosynthetic pathway, increasing the activity of MTPSs, and altering product profiles (Table 3). Furthermore, the detoxifying monoterpenoids, another key issue of monoterpenoids production in microbes, are briefly addressed here.

**Table 3:** Applications of plasticity engineering for microbial production of monoterpenoids

Applications	MTPSs/variants	Hosts	Substrates	Titer or major products	Ref.
Exploiting orthogonal pathway	MsLimS	*E. coli*	GPP	181.73 mg/L limonene	[74]
	MsLimS	*E. coli*	NPP	694.61 mg/L limonene	
	ClLimS2	*S. cerevisiae*	GPP	141.6 mg/L limonene	[73]
	ClLimS2	*S. cerevisiae*	NPP	917.7 mg/L limonene	
	ArLimS	*Y. lipolytica*	GPP	Limonene was undetected	[75]
	ArLimS	*Y. lipolytica*	NPP	23.56 mg/L limonene	
	ClLimS	*S. cerevisiae*	GPP	27.97 mg/L limonene	[31]
	ClLimS^H570Y^	*S. cerevisiae*	NPP	134.81 mg/L limonene	
	SpSabS	*S. cerevisiae*	GPP	17.67 mg/L sabinene	[31]
	SpSabS^H561F^	*S. cerevisiae*	NPP	72.39 mg/L sabinene	
Enhancing enzymatic activity	ObGerS	*S. cerevisiae*	GPP	8.4 mg/L geraniol	[77]
	ObGerS^F355Y^/ ObGerS^D507H^	*S. cerevisiae*	GPP	~ 10.7 mg/L geraniol	
	CrGerS	*S. cerevisiae*	GPP	54.8 mg/L geraniol	[78]
	CrGerS^F418Q^	*S. cerevisiae*	GPP	66.8 mg/L geraniol	
	MsLimS	*E. coli*	GPP	~ 542 mg/L limonene	[32]
	MsLimS^G566A−L571F^	*E. coli*	GPP	~ 940 mg/L limonene	
	PtPinS	*E. coli*	GPP	80 mg/L pinene	[79]
	PtPinS(PT1)^Q457L^	*E. coli*	GPP	150 mg/L pinene	
	PtPinS	Cyanobacteria	GPP	~ 40 μg/L pinene	[79]
	PtPinS(PT1)^Q457L^	Cyanobacteria	GPP	80 μg/L pinene	
Altering product spectrum	SeCamS	*S. cerevisiae*	GPP	~ 50% camphene	[31]
	SeCams^H583V^/ SeCams^H583F^	*S. cerevisiae*	NPP	Limonene predominantly	
	ClLimS	*S. cerevisiae*	GPP	99% C10 monoterpenoids	[61]
	ClLimS^H570V^/ ClLimS^H570L^/ ClLimS^H570I^	*S. cerevisiae*	2meGPP	70%–95% C11 terpenoids (69%–78% 2-methyllimonene of C11 products)	
	PtPinS(PT30)	*S. cerevisiae*	GPP	~ 75% C10 monoterpenoids	[61]
	PtPinS(PT30)^F607L^	*S. cerevisiae*	2meGPP	> 80% C11 terpenoids (56% 2-methyllinalool of C11 products)	
	PtPinS(PT30)^F607I^	*S. cerevisiae*	2meGPP	> 80% C11 terpenoids (75% 2-methylenebornane of C11 products)	

### Exploiting the orthogonal monoterpenoid biosynthetic pathway

The substrate engineering of plant MTPSs can be harnessed to enlarge the metabolic flux to product synthesis by exploiting a growth-orthogonal production module (Table 3). Microbes possess the native GPP-dependent terpenoid biosynthetic pathways to synthesize sterol for cell growth (Fig. 1), which are more competitive than the heterologous production of plant-derived monoterpenoids and decrease the metabolic flux to target products [73]. The plant NPP-dependent MTPSs have been proven to be efficient for the orthogonal monoterpenoid biosynthesis in both prokaryotic [74] and eukaryotic [31, 73, 75] host cells (Table 3). When using NPP other than GPP as the predominant substrate by MsLimS and ClLimS2, the production of limonene was increased by 282% and 548% in engineered *E. coli* [74] and *S. cerevisiae* [73], respectively. Furthermore, the NPP-dependent biosynthesis elevated the generation of sabinene by 43% and α-pinene by 169% in engineered yeast [31], respectively, indicating that exploiting the heterologous orthogonal biosynthetic pathway favored monoterpenoids production in microbes.

Considering the limited natural NPP-specific monoterpene synthases, plasticity engineering enables plant MTPSs to utilize NPP effectively. By engineering the scattered plasticity residues on the J/K loop, the NPP-specific ClLimS^H570Y^ and SpSabS^H561F^ were gained to synthesize limonene with a 382% increase and to produce sabinene with a 310% improvement in engineered yeast, respectively, compared to the wild-type enzymes with GPP as substrate [31].

### Enhancing monoterpene synthase activity

Modifying plasticity residues can significantly enhance the catalytic activity of plant MTPSs without changing substrate selectivity and product specificity and thus can boost the production of monoterpenoids in microbes (Table 3). Introducing double mutation (Q117H-T380I) to the *Cinnamomum osmophloeum* LinS augmented the catalytic activity up to 600% of the wild type in vitro [76]. Furthermore, one point mutation (F355Y, a scattered plasticity residue on helix F) of ObGerS increased geraniol production by 27% in *S. cerevisiae* [77]. Likewise, a homologous position mutation of F418Q of *Catharanthus roseus* GerS (CrGerS) boosted the production of geraniol by 21% in yeast [78]. Activity improvements by plasticity engineering are also embodied in the cyclic monoterpene synthases (Table 3). The double mutant MsLimS^G566A−L571F^ of Region 4 markedly increased the titer of limonene to 173% of the wild-type enzyme in *E. coli* [32]. Moreover, engineering the scattered plasticity residue Q457 of PtPinS(PT1) can change the metal cofactor preference from manganese to magnesium, which is three orders of magnitude richer in the cytosol [79]. Therefore, the mutant PtPinS(PT1)^Q457L^ showed a 100% improvement of pinene production in *E. coli* [79].

### Altering product spectrum

Plasticity engineering of plant MTPSs has been employed for altering the product spectrum in engineered microbes. The multipoint mutant MsLimS^S454G−C457V−M458I^ generated 62% pinene and 23% sabinene with the ratio of limonene being decreased from 96 to 4% in *E. coli*, and the variant LvFenS^T450G−C451G−T453V^ produced myrcene, limonene, terpinolene, and α-pinene with almost loss of fenchol (< 1%) in *E. coli* [32].

Plant MTPSs possess the likelihood of refining product profiles mediated by the utilization of different substrate isomers in microbes (Table 3). Compared to GPP, an array of acyclic plant MTPSs generated larger amounts of cyclic product limonene when using NPP as substrate [56, 80]. The distinct reaction mechanisms of MTPSs initiating from GPP and from NPP may contribute to the various product profiles. Different from NPP, GPP generates geranyl and linalyl cations first, thus increasing the probability to produce acyclic monoterpenoids (Fig. 2). Furthermore, the different binding modes of GPP or NPP in MTPSs can result in different products. The docked structure of MsLimS-NPP shows that the C7 atom of NPP is farther away from residues W324 and N345 than GPP, thus weakening the stabilization effect of polar residues in the active site cavity and leading to 22% bicyclic product pinene [72]. The wild-type SeCamS generated camphene as the major product in yeast with substrate GPP [31]. However, the SeCamS was converted to highly specific LimS by the modification of H583V or H583F (Table 3), which improved the selectivity for NPP to 300% of the wild-type enzyme [31].

Natural plant MTPSs can be engineered to produce non-natural terpenoids (Table 3). Mutants ClLimS^H570V^ and ClLimS^H570L^ were converted into C11-terpene synthases with 2-methyllimonene as the predominant product in the 2meGPP-producing yeast host, while variants PtPinS(PT30)^F607L^ and PtPinS(PT30)^F607I^ synthesized linear 2-methyllinalool and bicyclic 2-methylenebornane, respectively [61].

### Detoxifying monoterpenoids

Monoterpenoids are naturally defensive compounds for plants against pathogenic microorganisms and harmful insects [7]. Lipophilic monoterpenoids can interfere with the membrane functions of microorganisms [29, 81]. Linear and cyclic monoterpenoids may result in phase segregation and melting point depression in lipid bilayers [82], and they can increase the membrane fluidity and destroy the cellular integrity of microbes [83–85]. Furthermore, α-terpinene [85] and limonene [86] were reported to inhibit *S. cerevisiae* by damaging the cell wall. Monoterpenoid inhibition can also result from their induction of oxidative stress, which causes DNA damage or the formation of more toxic monoterpene hydroperoxide [29].

Detoxifying monoterpenoids to microorganisms has been paid more attention. Microbial chassis engineering can alleviate monoterpenoid toxicity and improve production [29], including exploiting efflux pumps to facilitate the release of monoterpenoids [87, 88] and improving cell membrane properties [89, 90] or stress response [29] to increase physiological tolerance. On the other hand, it is more serious that the high volatility of monoterpenoids causes the great loss of products in the fermentation process [91]. Therefore, an aqueous–organic two-phase system to extract products in situ is adopted as an effective strategy for monoterpenoids production due to the effects on capturing and detoxifying monoterpenoids [92]. The minimum inhibitory concentrations (MICs) of monoterpenoids are normally less than 600 mg/L against microbes [92, 93], but the MIC was increased by more than 700 times when using dibutyl phthalate as the organic phase for limonene production in *S. cerevisiae* [92]. Currently, biocompatible organic solvents, such as isopropyl myristate, diisononyl phthalate, and dodecane, have been extensively used in microbial production of monoterpenoids and other terpenoids [25, 27, 94–96]. Furthermore, the continuous in situ product removal techniques, which integrate the fermentation with the downstream recovery process, are more cost-effective and show a promising future for the production of terpenoids in microbes [97–99].

## Conclusions and future perspectives

In recent years, increasing reports focus on deciphering and exploiting the functional plasticity of plant MTPSs through protein engineering. Plant MTPSs contain four function-sensitive plasticity regions with a few plasticity residues scattering in the C-terminal domain, which influence the enzymatic properties through complex effects, including steric hindrance to the formation of carbocation intermediates, stabilization effect on the terpinyl cation, and control of the carbocation quenching by water molecules or electrostatic effect. By active site modifications or domain swapping, plasticity engineering of plant MTPSs can reconstruct the synthases to tolerate different substrate isomers, prenyl substrates of varying lengths, and even non-natural substrates. The product profiles can also be tamed among different degrees of structural complexity. However, the limited crystal structures restrict deep understanding of plant MTPSs. In future, resolving more three-dimensional structures of plant MTPSs, and the complex of MTPSs with authentic GPP or NPP substrates instead of fluorinated GPP or NPP with X-ray crystallography and electron cryo-microscopy, are required to further decipher the reaction cascades of the catalytic mechanisms and identify molecular determinants of functional plasticity.

With the advances of synthetic biology, microbial cell factories have been a promising alternative for terpenoids production. Plasticity engineering of plant MTPSs brings the dawn of success for constructing highly effective cell factories for monoterpenoids production. The production titers can be elevated by harnessing the effective NPP-dependent orthogonal biosynthetic pathway, or by boosting the enzymatic activity directly. It has also been advanced to alter the product spectra of plant MTPSs and expand the territory of terpenoids to non-natural terpenoids. However, the application of the plasticity engineering of plant MTPSs remains challenging. Random modification of synthases is fairly labor-intensive and time-consuming, while rational engineering provides a more reliable alternative [100]. Selecting residues for modification from plasticity regions of plant MTPSs or according to the results of molecular dynamics simulations, the functional changes of substitutions can be more predictable [101]. Furthermore, customizing the Design–Build–Test–Learn cycle by high-throughput screening method for monoterpenoids, such as developing an automatic pipeline [102], can significantly enhance the efficiency and fuel the application of plasticity engineering of plant MTPSs for microbial production of terpenoids.

## Supplementary Information

**Additional file 1: Table S1.** Key applications of important monoterpenoids. **Table S2.** Plant monoterpene synthases and corresponding Uniprot entry that have been discussed in this review.

## Data Availability

Not applicable.

## Abbreviations

MEP: Methylerythritol-4-phosphate
MVA: Mevalonate
DMAPP: Dimethylallyl diphosphate
IPP: Isopentenyl diphosphate
GPP: Geranyl diphosphate
NPP: Neryl diphosphate
FPP: Farnesyl diphosphate
GGPP: Geranylgeranyl diphosphate
LPP: Linalyl diphosphate
BPP: Bornyl diphosphate
2meGPP: 2-Methyl-geranyl diphosphate
SAM: S-Adenosyl methionine
IDI: Isopentenyl diphosphate isomerase
GPPS: Geranyl diphosphate synthase
NPPS: Neryl diphosphate synthase
FPPS: Farnesyl diphosphate synthase
GGPPS: Geranylgeranyl diphosphate synthase
TPS: Terpene synthase
MTPS: Monoterpene synthase
MyrS: Myrcene synthase
GerS: Geraniol synthase
LinS: Linalool synthase
LimS: Limonene synthase
TerS: Terpinene synthase
TepS: Terpineol synthase
PinS: Pinene synthase
BPPS: Bornyl diphosphate synthase
CamS: Camphene synthase
SabS: Sabinene synthase
CarS: 3-Carene synthase
FenS: Fenchol synthase
CinS: 1,8-Cineole synthase
