# Supplementary material for: Plasticity engineering of plant monoterpene synthases and application for microbial production of monoterpenoids
Source: Biotechnol Biofuels. 2021 Jun 30;14:147. doi: 10.1186/s13068-021-01998-8 (PMC8247113; doi:10.1186/s13068-021-01998-8)
Supplement: Supplementary file 1 — Additional file 1: Table S1. Key applications of important monoterpenoids. Table S2. Plant monoterpene synthases and corresponding Uniprot entry that have been discussed in this review. [file 13068_2021_1998_MOESM1_ESM.docx]

**Supplementary information**

Plasticity engineering of plant monoterpene synthases and application for microbial production of monoterpenoids

Dengwei Lei^1^, Zetian Qiu^1^, Jianjun Qiao^1,2^, Guang-Rong Zhao^1,2*^

^1^Frontier Science Center for Synthetic Biology and Key Laboratory of Systems Bioengineering (Ministry of Education), School of Chemical Engineering and Technology, Tianjin University, Yaguan Road 135, Jinnan District, Tianjin, 300350, China.

^2^SynBio Research Platform, Collaborative Innovation Centre of Chemical Science and Engineering (Tianjin), Tianjin University, Yaguan Road 135, Jinnan District, Tianjin, 300350, China.

Keywords: monoterpene synthase, functional plasticity, synthetic biology, enzyme engineering, substrate selectivity, product specificity, monoterpenoid production.

*Corresponding author: grzhao@tju.edu.cn

Tel: +86-22-85356580; Fax: +86-22-27403389

**Table S1.** Key applications of important monoterpenoids.

| Monoterpenoids | Classes | Key applications | References |
| --- | --- | --- | --- |
| Myrcene | Acyclic monoterpene | As a ﬂavor ingredient in foods and as a fragrance in cosmetics and cleaning products;  As a renewable compound for producing fine chemicals. | [1,2] |
| Linalool | Acyclic monoterpene alcohol | As a fragrance in cosmetics and cleaning products;  As a potential repellent;  As a precursor for the jet fuel RJ‐4. | [3,4,5] |
| Geraniol | Acyclic monoterpene alcohol | As a ﬂavor ingredient in foods and as a fragrance in cosmetics;  As a potential insecticide. | [3,6] |
| Nerol | Acyclic monoterpene alcohol | As a ﬂavor ingredient in foods and as a fragrance in cosmetics and cleaning products. | [7] |
| Limonene | Monocyclic monoterpene | As a ﬂavor ingredient in foods and as a fragrance in cosmetics and cosmetics;  As a potential insecticide;  For the treatment of cholecystitis and angiocholitis; Limonene dimer can be used as jet fuel. | [8,9,10] |
| β-Phellandrene | Monocyclic monoterpene | As an ingredient in cosmetics and cleaning products;  Potentially as a fuel. | [11] |
| α-Terpineol | Monocyclic monoterpene alcohol | As a ﬂavor ingredient in foods and as a fragrance in cosmetics and cosmetics. | [12] |
| Paeoniflorin | Glycosylated derivative of limonene | Potentially for the treatment of ischemic strokes. | [13] |
| Pinene | Bicyclic monoterpene | Pinene dimer can be used as jet fuel. | [10] |
| Sabinene | Bicyclic monoterpene | As a biofuel. | [14,15] |
| Borneol | Bicyclic monoterpene alcohol | As a ﬂavor ingredient in foods and as a fragrance in cosmetics and cleaning products;  As an analgesic drug for treating burns, wounds, cuts, and injuries, and as an indispensable ingredient of traditional Chinese medicines for cardiovascular diseases, including stroke, angina pectoris, and coronary heart disease. | [16,17] |
| 1,8‐Cineole | Bicyclic monoterpene alcohol | Can be used to treat cardiovascular illness, digestive sickness, Alzheimer’s disease, and respiratory ailment;  As a precursor of p‐cymene for producing the biojet fuel blend AMJ‐700. | [18,19] |

**Table S2.** Plant monoterpene synthases and their Uniprot entry discussed in this review.

| Abbreviations | Monoterpene synthases | Organisms | Uniprot entry |
| --- | --- | --- | --- |
| ObMyrS | Myrcene synthase | *Ocimum basilicum* | Q5SBP1 |
| ObGerS | Geraniol synthase | *Ocimum basilicum* | Q6USK1 |
| PcGerS | Geraniol synthase | *Perilla citriodora* | Q4JHG3 |
| CrGerS | Geraniol synthase | *Catharanthus roseus* | J9PZR5 |
| PhLinS | Linalool synthase | *Perilla hirtella* | C0KWV5 |
| MsLimS | Limonene synthase | *Mentha spicata* | Q40322 |
| CsLimS | Limonene synthase | *Citrus sinensis* | A0A1C9J6A7 |
| ClLimS | Limonene synthase | *Citrus limon* | Q8L5K3 |
| ClLimS2 | Limonene synthase | *Citrus limon* | Q8L5K1 |
| AgLimS | Limonene synthase | *Abies grandis* | O22340 |
| ArLimS | Limonene synthase | *Agastache rugosa* | Q940E7 |
| TvTerS | γ-Terpinene synthase | *Thymus vulgaris* | A0A0M3Q1Q3 |
| AgLim/PinS | Limonene/α-pinene synthase | *Abies grandis* | Q9M7C9 |
| TcTeo/PinS | α-Pinene/terpinolene synthase | *Taiwania cryptomerioides* | A0A0A7DLU1 |
| AgPinS | Pinene synthase | *Abies grandis* | O24475 |
| PtPinS(PT30) | Pinene synthase | *Pinus taeda* | Q84KL3 |
| PtPinS(PT1) | Pinene synthase | *Pinus taeda* | Q84KL6 |
| SoBPPS | Bornyl diphosphate synthase | *Salvia officinalis* | O81192 |
| SeCamS | Camphene synthase | *Solanum elaeagnifolium* | Not found |
| SoSabS | Sabinene synthase | *Salvia officinalis* | O81193 |
| PsSabS | Sabinene synthase | *Picea sitchensis* | F1CKJ1 |
| PsCarS2 | 3-Carene synthase | *Picea sitchensis* | F1CKI8 |
| LvFenS | Fenchol synthase | *Lavandula viridis* | T1RR72 |
| SoCinS | 1,8-Cineole synthase | *Salvia officinalis* | O81191 |
| SfCinS | 1,8-Cineole synthase | *Salvia fruticosa* | A6XH05 |

# References

1. Behr A, Johnen L. Myrcene as a natural base chemical in sustainable chemistry: a critical review. ChemSusChem. 2009;2:1072-1095.
2. Hwang E, Ngo HTT, Park B, Seo SA, Yang JE, Yi TH. Myrcene, an aromatic volatile compound, ameliorates human skin extrinsic aging via regulation of MMPs production. Am J Chin Med. 2017;45:1113-1124.
3. Xie SS, Zhu L, Qiu XY, Zhu CS, Zhu LY. Advances in the metabolic engineering of *Escherichia coli* for the manufacture of monoterpenes. Catalysts. 2019;9:433.
4. Kamatou G, Viljoen AM. Linalool--a review of a biologically active compound of commercial importance. Nat Prod Commun. 2008;3:1183-1192.
5. Meylemans HA, Quintana RL, Goldsmith BR, Harvey BG. Solvent-free conversion of linalool to methylcyclopentadiene dimers: a route to renewable high-density fuels. ChemSusChem. 2011;4:465-469.
6. Dehsheikh AB, Sourestani MM, Dehsheikh PB, Mottaghipisheh J, Vitalini S, Iriti M. Monoterpenes: essential oil components with valuable features. Mini Rev Med Chem. 2020;20:958-974.
7. Lapczynski A, Foxenberg RJ, Bhatia SP, Letizia CS, Api AM. Fragrance material review on nerol. Food Chem Toxicol. 2008;11:S241-244.
8. Ciriminna R, Lomeli-Rodriguez M, Cara PD, Lopez-Sanchez JA, Pagliaro M. Limonene: a versatile chemical of the bioeconomy. Chem Commun. 2014;50:15288-15296.
9. Wang S, Chen Y, Gao Z, Xiong M, Zhong Z, Ye L. Gas chromatographic-mass spectrometric analysis of d-limonene in human plasma. J Pharm Biomed Anal. 2007;44:1095-1099.
10. Meylemans HA, Quintana RL, Harvey BG. Efficient conversion of pure and mixed terpene feedstocks to high density fuels. Fuel. 2012;97:560-568.
11. Formighieri C, Melis A. Carbon partitioning to the terpenoid biosynthetic pathway enables heterologous β-phellandrene production in *Escherichia coli* cultures. Arch Microbiol. 2014;196:853-861.
12. Sales A, Felipe LDO, Bicas JL. Production, properties, and applications of α-terpineol. Food Bioproc Tech. 2020;13:1261-1279.
13. Zhang Y, Li H, Huang M, Huang M, Chu K, Xu W, Zhang S, Que J, Chen L. Paeoniflorin, a monoterpene glycoside, protects the brain from cerebral ischemic injury via inhibition of apoptosis. Am J Chin Med. 2015;43:543-557.
14. Rude MA, Schirmer A. New microbial fuels: a biotech perspective. Curr Opin Microbiol. 2009;12:274-281.
15. Peralta-Yahya PP, Keasling JD. Advanced biofuel production in microbes. Biotechnol J. 2010;5:147-162.
16. Bhatia SP, Letizia CS, Api AM. Fragrance material review on borneol. Food Chem Toxicol. 2008;11:S77-80.
17. Yang Z, An W, Liu S, Huang Y, Xie C, Huang S, Zheng X. Mining of candidate genes involved in the biosynthesis of dextrorotatory borneol in *Cinnamomum burmannii* by transcriptomic analysis on three chemotypes. PeerJ. 2020;8:e9311.
18. Cai ZM, Peng JQ, Chen Y, Tao L, Zhang YY, Fu LY, Long QD, Shen XC. 1,8-Cineole: a review of source, biological activities, and application. J Asian Nat Prod Res. 2020. https://doi.org/10.1080/10286020.2020.1839432.
19. Mendez-Perez D, Alonso-Gutierrez J, Hu Q, Molinas M, Baidoo EEK, Wang G, Chan LJG, Adams PD, Petzold CJ, Keasling JD, et al. Production of jet fuel precursor monoterpenoids from engineered *Escherichia coli*. Biotechnol Bioeng. 2017;114:1703-1712.
